# Advancements in understanding substantia nigra hyperechogenicity via transcranial sonography in Parkinson’s disease and its clinical implications

**DOI:** 10.3389/fneur.2024.1407860

**Published:** 2024-07-18

**Authors:** Yuan-yuan Zhang, Xu-hong Jiang, Pei-pei Zhu, Wen-yan Zhuo, Li-bin Liu

**Affiliations:** ^1^Department of Neurology, Zhuhai People’s Hospital, Zhuhai, Guangdong, China; ^2^Department of Health Management, Zhuhai People’s Hospital, Zhuhai, Guangdong, China

**Keywords:** transcranial sonography, abnormal hyperechogenicity, mechanisms, clinical relevance, Parkinson

## Abstract

Amidst rising Parkinson’s disease (PD) incidence in an aging global population, the need for non-invasive and reliable diagnostic methods is increasingly critical. This review evaluates the strategic role of transcranial sonography (TCS) in the early detection and monitoring of PD. TCS’s ability to detect substantia nigra hyperechogenicity offers profound insights into its correlation with essential neuropathological alterations—namely, iron accumulation, neuromelanin depletion, and glial proliferation—fundamental to PD’s pathophysiology. Our analysis highlights TCS’s advantages, including its non-invasiveness, cost-effectiveness, and ease of use, positioning it as an invaluable tool for early diagnosis and continual disease progression monitoring. Moreover, TCS assists in identifying potential risk and protective factors, facilitating tailored therapeutic strategies to enhance clinical outcomes. This review advocates expanding TCS utilization and further research to maximize its diagnostic and prognostic potential in PD management, contributing to a more nuanced understanding of the disease.

## Introduction

Parkinson’s disease (PD) is a common central nervous system disease in middle-aged and elderly people and is the second-most degenerative disease of the nervous system after Alzheimer’s disease. According to Wirdefeldt et al. (1), 1% of people over 60 years of age are diagnosed with PD. An epidemiological survey showed that its prevalence in the population over 65 years was close to 1.7% (2). The age of onset of PD is mostly over 50 years, and the incidence is slightly higher incidence in males than females. The main pathological features of PD are the loss of dopaminergic neurons and the formation of Lewy bodies in the substantial nigra (SN) pars compacta (SNc) (3). At present, the diagnosis of PD still mainly depends on clinical manifestations. The assessment of disease stage and severity also mainly relies on the subjective evaluation of clinicians, with few objective auxiliary examination methods (4). The current diagnostic criteria for PD are based on the diagnostic criteria for PD in the UK Brain Bank and were developed with reference to the clinical diagnostic criteria for PD launched by the International Parkinson and Movement Disorder Society in 2015 (5). Since Becker et al. (6) first reported that transcranial sonography (TCS) through the temporal window could detect the characteristic manifestation of SN hyperechogenicity in patients with PD in 1995, an increasing number of scholars in China and other countries have carried out applied research on PD through TCS. In this paper, we focus on the mechanisms and clinical relevance of the emergence of SN hyperechogenicity arises in patients with PD.

### TCS examination of patients with PD showed that SN hyperecho was related to iron deposition

It is not well understood how SN hyperechogenicity on TCS arises in patients with PD. Existing studies mainly focus on iron ion deposition, reductions in neuromelanin (NM), and glial cell hyperplasia (7–9). Patients with PD have abnormal iron deposition in the brain (10), and the accumulated iron undergoes the Fenton reaction (in which ferrous salts and hydrogen peroxide combine to form hydroxyl free radical, which has a powerful oxidative degradation effect) and the Haber-Weiss reaction (in which hydrogen peroxide is converted to hydroxyl free radicals under the action of ferrous ions) and will produce a large amount of strongly oxidizing hydroxyl free radical. This hydroxyl radical causes oxidation of neural membrane lipids, causing disruption of dopamine nerve cell membrane function and ultimately progressive loss of dopaminergic neurons (11). At the same time, pathological iron deposition can induce α-synuclein accumulation, over expression, and abnormal folding that can lead to the generation of Lewy bodies, giving rise to PD (12). Acosta-Cabronnero et al. (13) conducted a quantitative susceptibility mapping (QSM) (a recently developed magnetic resonance imaging technique based on susceptibility-weighted imaging sequences that can accurately quantify iron deposition in patients’ brains) study that included 25 patients with PD and 50 healthy controls (14). They showed that patients with PD had significantly more iron deposition in the SNc than the control group, and the abnormal region of iron deposition in the brain coincided with the region of ɑ-synuclein distribution. In recent years, QSM studies of patients with PD have found an abnormal increase in iron deposition in SN, suggesting a strong association between PD and iron deposition in the brain, especially in SN (15). Azuma et al. (16) applied QSM to compare 24 patients with PD and 24 healthy controls. After categorizing the degree of bilateral limb involvement in the disease course, found that the magnetic susceptibility (an indicator of QSM that shows a linear relationship with iron deposition in the brain) of the SN on the severely affected side was higher than the mildly affected side and control group, and the iron deposition in the SN of PD patients was significantly higher compared to the healthy control group. Another study showed that this change in iron deposition has already occurred by the early stages of PD. In a study that included 60 patients with PD and 40 healthy controls, according to the Hoehn & Yahr (H-Y) stage, Guan et al. (17) divided patients with PD into the early-stage group (H-Y stage ≤2.5) and the late-stage group (H-Y stage ≥3). The iron deposition in SN was significantly higher in both early-stage PD and late-stage PD groups compared to the control group. Additionally, in the late-stage PD group, the iron deposition level in the SNc was higher compared to both the control and early-stage PD group. In addition, studies by He et al. (18) and An et al. (19) found that the Unified Parkinson’s Disease Rating Scale (UPDRS) total score (UPDRS score indicates a total of 199 points) and UPDRS-III score (UPDRS-III scores a total of 56 points) were significantly higher in late-stage patients with PD than early-stage patients with PD, and UPDRS-III score was positively correlated with iron deposition in SN. That is, the more pronounced the motor symptoms in patients with PD, the higher the iron deposition in SN, which again confirmed that PD is closely related to iron deposition in SN and that there is a correlation between iron deposition and disease severity.

Iron plays a crucial role in many biological processes, including oxygen transportation, DNA synthesis, and electron transport in neurons. However, excessive iron accumulation can be toxic and is associated with oxidative stress and neuronal damage (20). This oxidative stress is thought to contribute to the degeneration of dopaminergic neurons observed in PD (20). In the study by Zecca et al. (14), TCS was utilized to measure the echogenic area of the SN region and analyze its correlation with the concentrations of iron, H-ferritin, and L-ferritin. The results indicated a significant positive correlation between the echogenic area of the SN and the concentrations of iron and its associated proteins (14). The hyperechogenicity observed could be partly due to the increased iron deposition which reflects ultrasound waves more than the surrounding tissues (21). Ceruloplasmin’s ferroxidase activity is essential for preventing iron accumulation in the brain (22). Lower activity leads to increased levels of ferrous iron within the neurons, promoting oxidative stress and contributing to the dopaminergic neuron loss characteristic of PD. TCS can sensitively detect pathological metal accumulation (23). Berg et al. (24) found that injection of iron and 6-hydroxydopamine (6-OHDA) (a destructive agent of dopaminergic cells that can release iron from ferritin) enhanced the echogenicity of SN in a dose-dependent manner. In 2006, their team autopsied the brain tissues of 60 patients with PD of different ages and confirmed that the echogenic characteristics of the SN were positively correlated with iron deposition in the SN and were not correlated with other heavy metal levels (metals with densities above 4.5 g/m^3^), such as copper (25).

SN hyperechogenicity is not unique to PD. SN hyperechogenicity can be detected in 90% of patients with PD as well as in approximately 8–14% of healthy individuals (26). In normal populations, iron deposition most often occurs during adolescence and early adulthood. The structures with the highest iron content in brain tissue are the globus pallidus and SN, which gradually decrease in adulthood. A study of 109 newborns showed that the SN region of most newborns exhibits significantly elevated echogenic on TCS, which diminish gradually with age. By around the age of 16, the echogenic level in the SN approaches that of adults. This suggests that iron metabolism and brain tissue development have reached maturity at this stage (27). A study of 330 healthy subjects showed that 8–9% of the subjects showed the phenomenon of SN hyperechogenicity. Positron-emission tomography of 10 subjects with SN hyperechogenicity revealed a significant decrease in striatal dopamine uptake, indicating that although there were no signs or symptoms of PD, the subjects with enhanced SN echogenicity may have subclinical or preclinical striatum damage (28). We speculate that the SN hyperechogenicity in adolescence and early adulthood may suggest an increased susceptibility to SN injury if the SN hyperechogenicity does not undergo a normal physiological decline.

### TCS examination of patients with PD showed that SN hyperecho was associated with NM reduction

A multivariate analysis of TCS data (14) showed that SN echo intensity and area were negatively correlated with NM content. NM is an insoluble brown pigment composed of covalently bound melanin groups, lipids, and peptides. It is a byproduct of catecholamine oxidative catabolism and is present in a variety of neurons, mainly in the dopaminergic neurons of the SN (28). PD is a neurodegenerative disease characterized by selective loss of NM-containing neurons in SNc and the appearance of Lewy bodies. Pathological studies have confirmed that the NM of the SNC is significantly lower in patients with PD (29, 30). NM can reduce the oxidative damage of cells by removing excessive cytosolic dopamine and chelated iron and avoid the oxidative stress response in brain tissue (31, 32). In contrast, patients with PD have a significant loss of NM content in the SN compared to normal subjects (30). A decrease in the ability of NM to chelate iron leads to an accumulation of free iron in the cell, which in turn leads to an increase in the degree of oxidative damage in the cell.

### TCS examination of patients with PD showed that SN hyperecho was associated with gliosis

In an autopsy study (33), Berg found that the echogenic properties of the SN were related to the activation of microglia after correction of iron and NM content, showing that microglial activation was an independent factor influencing the changes in SN echogenicity. Additionally, astrocytes also play an important role in SN echogenicity alterations. It is widely accepted that the PD-related glial cells are mainly astrocytes and microglia. Astrocytes are the most abundant cell type in the central nervous system, and play a dual role in the occurrence and progression of PD. On the one hand, glial-derived neurotrophic factors secreted by astrocytes can promote the survival and morphological differentiation of midbrain dopaminergic neurons, and increase their affinity for dopamine uptake, thereby mitigating the pathological progression of PD. On the other hand, in PD patients, astrocytes gathered a large number of α-synuclein proteins, these proteins are likely to be toll-like receptors and trigger inflammation upon recognition by molecular ligands, potentially leading to further damage and death of dopaminergic (34–37).

Microglia are innate immune cells that protect neurons from harmful stimuli (38). They account for 12% of the cells in the entire central nervous system. They are distributed throughout the brain parenchyma and form the first line of immune defense in the nervous system. After proliferation and activation, they can engulf cell debris and the myelin sheath of the lesion, eliminating dead cells and degenerative material and promoting tissue repair (39).

Cicchetti et al. (40) used a stereotactic technique to inject neurotoxin 6-OHDA into the right striatum of rats to create a PD animal model and found that the number of right SN dopaminergic neurons was significantly reduced. At the same time, the numbers of astrocytes and microglia significantly increased. Tumor necrosis factor-alpha (TNF-α) was positively expressed in the right SN and striatum of the model group, and it was mainly distributed on activated microglia. In the 1-methyl-4-phenyl-1,2,3,6-tetrahydropyridine (MPTP)-induced mouse PD model, the degree of loss of dopaminergic neurons was parallel to the activation of microglia at this site (40). An insecticide, rotenone, can cause symptoms and pathological changes similar to PD in rodents, and local microglial activation has been observed without exception in the rotenone-exposure animal model (41). Glial fibrillary acidic protein (GFAP) is a marker of astrocyte activation. GFAP expression was significantly increased in the autopsy of PD patients and in the brain tissue of PD animal models. This result suggests that many activated proliferative astrocytes are active in the pathological process of PD (42). The injection of lipopolysaccharide into the SNc of male mice can cause the activation of microglia and produce a large amount of interleukin-1β (IL-1β) (43). Injecting NM into the SN of rats, it was also observed that extracellular NM rapidly induced the activation of microglial. Activated microglia produced large amounts of oxidative stressors and pro-inflammatory factors, thereby inducing dopaminergic neurodegeneration in patients with PD (44). The above studies indicate that the neuroinflammatory response induced by the activation of microglia and astrocytes plays a decisive role in the occurrence and progression of PD.

TNF-α is mainly produced by activated macrophages, NK cells and T lymphocytes. TNF-α can stimulate cerebrovascular endothelial cells to express adhesion factors and activate glial cells to produce more inflammatory mediators, thus stimulating the body’s local disease response. Significantly elevated IL-1β and other inflammatory factors have been found in cerebrospinal fluid and serum of patients with PD, but the manifestations of different inflammatory factors are different in cerebrospinal fluid and serum, and the increase of serum IL-1β indicates the rapid development of PD (45). Studies have found that the injection of 6-OHDA into the striatum induced PD rat model can reduce the inflammation of injured striatal neurons by inhibiting the expression of IL-1β and other inflammatory cytokines (46). Some studies have also pointed out that lipopolysaccharide activates microglia and induces neuroinflammation by accelerating the expression of various pro-inflammatory cytokines such as IL-1β (47). Studies have shown that the occurrence and development of PD may be due to the cytotoxic effect caused by the increased expression of IL-1β, which leads to the chronic degeneration and even death of neurons (45). IL-6 is a cytokine. It acts on the blood–brain barrier, altering its permeability in such a way that peripheral immune cells can penetrate into the center, activate glial cells to produce inflammatory factors, and induce or exacerbate neurodegenerative disease (48, 49).

**Figure 1:**
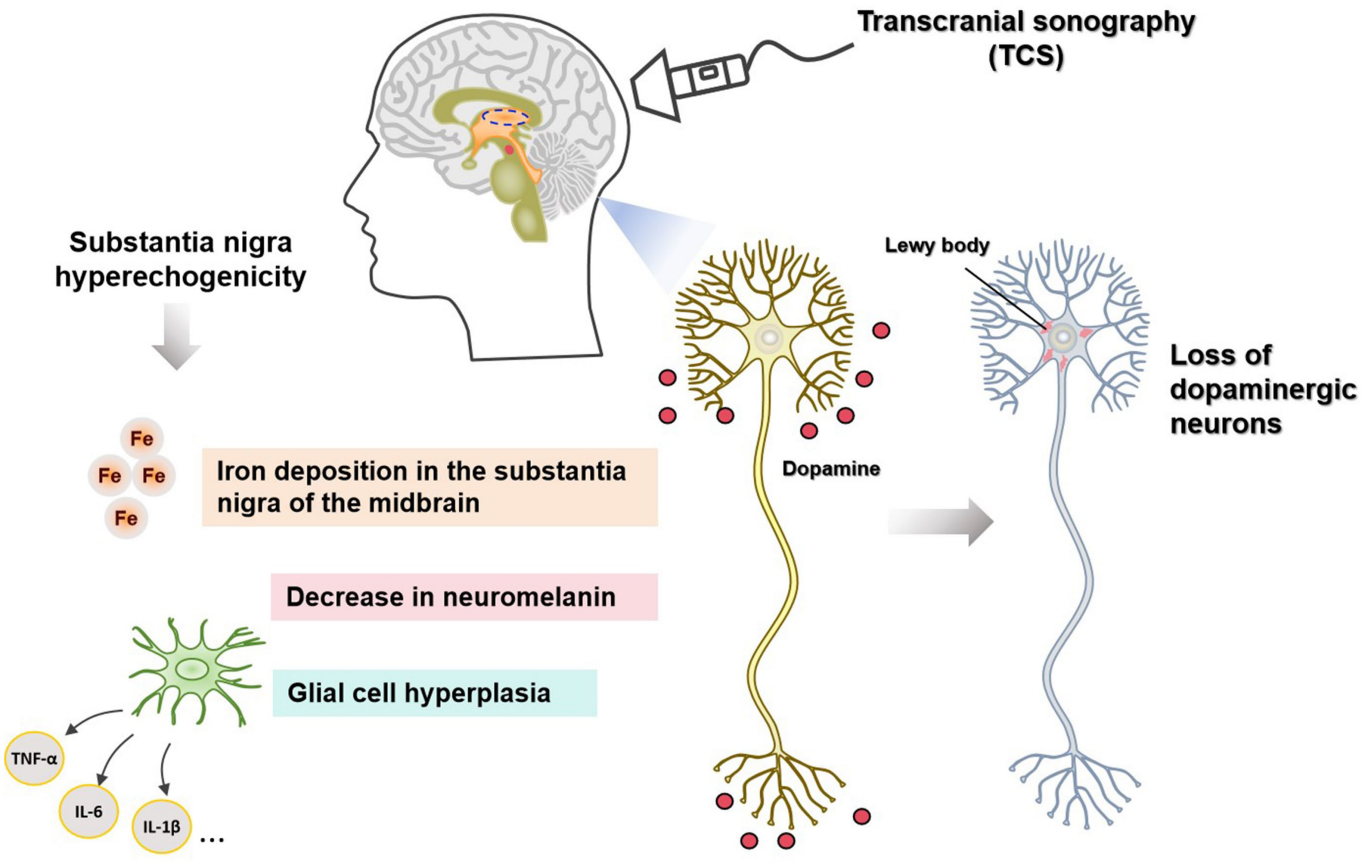
Mechanisms of substantial nigra (SN) hyperecho generation on the transcranial sonography (TCS) in patients with Parkinson’s disease.

Studies on the mechanism of SN hyperechogenicity (shown in Table 1, Figure 1) have shown a close connection between SN hyperechogenicity and PD pathophysiology. Understanding the mechanism of SN hyperechogenicity and its relationship with the pathophysiological changes of PD may provide new ideas for the clinical treatment of PD. Reducing iron deposition in SN, upholding the intracellular levels of NM, and antagonizing glial cell proliferation may delay the pathological changes in PD and the occurrence of neurological deficit symptoms.

**Table 1:** The mechanism of SN in PD patients.

Factors	Mechanism of substantia nigra hyperechogenicity in PD patients
Iron deposition in the substantia nigra of the midbrain	The accumulated iron, through Fenton reaction and Haber-Weiss reaction, produces a large number of hydroxyl radicals with strong oxidation, which participate in oxidative stress response and eventually cause the progressive loss of dopaminergic neurons.
Decrease in neuromelanin	Substantia nigra hyperechogenicity is associated with damage to the corticostriatum, manifested by the loss of dopamine neurons, which are rich in neuromelanin, and the substantia nigra appears hyperechogenicity when dopamine neurons in the substantia nigra are involved.
Glial cell hyperplasia	The activation of glial cells releases biologically active molecules, such as inflammatory cytokines such as IL-1β, IL-6, TNF-α, chemokines, anti-inflammatory factors and neuromodulin. These active substances cause chronic and persistent inflammatory response, leading to the degenerative loss, degeneration and even necrosis of DA neurons.

## The association between SN hyperechogenicity and PD subtypes

Recent research in the field of PD has increasingly focused on the varied manifestations of the disease, particularly the distinctions between the postural instability gait difficulty (PIGD) and tremor dominant (TD) subtypes (50, 51). Transcranial sonography (TCS), which detects hyperechogenicity of the substantia nigra (HSN), presents a valuable tool in exploring these differences. HSN has been correlated with various pathological and clinical features of PD, suggesting that it may play a role in distinguishing PD subtypes. Studies have indicated that HSN is more frequently observed and tends to be more pronounced in patients diagnosed with the PIGD subtype as compared to the TD subtype (52). This difference may reflect the underlying pathophysiological variations between these forms of PD. For example, patients with PIGD tend to exhibit a greater overall neurodegenerative burden, including more extensive loss of dopaminergic neurons and higher levels of iron deposition in the substantia nigra. These factors contribute to the increased echogenicity observed via TCS (53). Furthermore, the prevalence and intensity of HSN in PIGD patients have been associated with more severe motor dysfunctions and a faster disease progression, underscoring the clinical relevance of TCS as a diagnostic and prognostic tool (54). This imaging technique could potentially aid clinicians in more effectively anticipating disease trajectory and tailoring interventions. Conversely, the TD subtype shows a less consistent association with HSN. Some studies suggest a lower occurrence of HSN among TD patients, which may align with the generally slower progression rate and milder motor impairment observed in this group (55). The differential findings in HSN prevalence between PIGD and TD subtypes underscore the importance of considering subtype-specific pathologies in PD diagnosis and management. This emerging evidence suggests that TCS, through the assessment of HSN, may become an integral part of the diagnostic process, helping to differentiate between PD subtypes at an early stage. It also highlights the need for further research to elucidate the mechanisms driving the differences in substantia nigra hyperechogenicity and their implications for PD subtype diagnosis and treatment strategies.

### Clinical relevance of the use of TCS for SN hyperechogenicity in patients with PD

In more than 90% of patients with PD, TCS indicates that SN hyperechogenicity is a typical and stable feature. Clinical correlation studies on SN echo characteristics (including echo intensity and area size) have lacked the support of large samples. Few clinical indicators were included in the study. In addition to the factors that have been discussed such as sex, age, onset mode, disease course, and disease severity, other clinical factors such as plasma uric acid, blood lipids, vitamin D, and homocysteine, may also be associated with SN hyperechogenicity. However, large-scale, multicenter studies are needed to be confirm theses associations.

### Correlation with sex, age, onset pattern, disease course, and disease severity

Various studies have reached inconsistent conclusions on the correlation between SN hyperechoic characteristics and sex, age, mode of onset, and disease severity. Becker et al. (6) analyzed and compared 30 patients with PD and 30 healthy controls and found that SN hyperechogenicity was associated with disease severity. In 2010, a case–control study involving 115 subjects found that the SN hyperechoic area was larger in patients with onset of akinetic-rigid and bradykinesia, and the SN hyperechoic characteristics were correlated with age but not with sex (56). Joana et al. (57) showed that the SN hyperechogenicity was not associated with the severity of the disease. In 2017, a study of 373 patients with PD also found that akinetic-rigid or mixed PD had a larger SN hyperechoic area than tremor-dominant PD, but the SN hyperechogenicity was not correlated with the disease course or disease severity (55). Toomsoo et al. (58) analyzed 300 patients with PD and 200 healthy controls and found that the SN hyperechoic area of middle-aged and elderly patients with PD was smaller than that of young patients with PD, and the SN echo area was positively correlated with age in healthy people. In another cross-sectional study involving 374 patients with PD, SN echo characteristics were positively correlated with sex, age, age of onset, and disease severity (9). In summary, the correlation between SN hyperechogenicity and sex, age, onset pattern, and disease severity still require more study to draw true and reliable conclusions.

### Correlation with plasma uric acid level

In a meta-analysis that included 2,379 patients with PD and 2,367 controls, Wen et al. (59) found that the plasma uric acid level of patients with PD was significantly lower than that of healthy people. Plasma uric acid levels were lower in patients with PD with intermediate to advanced H-Y stage, indicating a negative association between plasma uric acid levels and H-Y stage. Another study corroborated their findings (60). A randomized controlled trial applied the uric acid precursor inosine to patients with PD and found that oral administration **of** inosine effectively increased the plasma uric acid level and delayed the progression of PD, further verifying the above conclusions (43). Uric acid is an important antioxidant and free radical scavenger in the human body that chelates iron. It lowers specific iron deposition in the SN region of patients with PD and plays a protective role in dopaminergic neurons. Therefore, a decrease in serum uric acid level may affect iron deposition and increase iron deposition in the SN area, thereby showing high echogenicity in TCS (61). Uric acid also acts as a scavenger of peroxynitrite, blocking nitrite-mediated nitration and preventing damage to cells caused by nitration of the protein tyrosine. It can be seen that a certain level of uric acid can reduce the oxidative stress damage of cells to a certain extent. Oxidative stress is one of the important pathogeneses of PD, and uric acid is likely to exert its neuroprotective effect by reducing the level of oxidative stress in PD (62).

Abnormal blood lipid metabolism (63, 64), plasma 25-hydroxyvitamin D levels (65–67), and plasma homocysteine levels (59, 68, 69) are associated with the occurrence and progression of PD. There have been few studies on whether these biochemical indicators are related to the characteristic SN echogenicity. The relevant factors affecting SN echogenicity in PD patients were analyzed and summarized to identify risk factors and protective factors. The results provide a theoretical basis for early intervention and individualized treatment of relevant risk factors in clinical practice.

## Discussion and summary

The review explored TCS as a non-invasive method to detect abnormal hyperechogenicity in the substantia nigra of patients with PD, offering a comparison with other imaging techniques like MRI and CT scans, and discussed how the hyperechoic features of the substantia nigra, as revealed by TCS, correlate with various clinical indicators such as age, disease progression, severity, and certain biochemical markers. The review concluded that TCS can serve as a valuable tool for early intervention and personalized treatment strategies by identifying risk factors and protective factors for PD.

In analyzing the factors contributing to SN hyperechogenicity detected by TCS, we first emphasized the crucial role of iron deposition in the pathogenesis of PD. The echogenic properties of the SN are closely associated with iron deposition (15), highlighting its potential as a diagnostic and monitoring marker in TCS. Additionally, NM protects dopaminergic neurons through its antioxidant properties and ability to chelate iron ions. In PD, the reduction of NM leads to the accumulation of iron ions and increased oxidative stress, exacerbating neuronal degeneration. The echogenic characteristics of the SN reflect changes in iron deposition and NM content (30). Furthermore, we observed that the activation of microglial cells plays a key role in the pathological process of PD. Activated microglia release a substantial amount of oxidative stress products and pro-inflammatory factors, contributing to neuronal damage. While microglia can promote neuronal repair by phagocytosing cellular debris and removing damaged substances, dysregulated activation may result in chronic inflammation and neurotoxicity, thereby accelerating the progression of PD (34, 44).

In the final section of the review, we discussed the clinical significance of TCS and derived therapeutic insights based on the aforementioned detection mechanisms. These insights suggest that TCS could be a valuable tool not only for diagnosing PD but also for informing personalized treatment strategies. TCS can be used to demonstrate the characteristic structural changes of the SN in PD patients by using the principle of ultrasound imaging. The sensitivity of TCS in the diagnosis of PD is between 91 and 100% (50, 51). In 2013, the European Federation of Neurological Societies and the Movement Disorders Society recommended the finding of SN echogenic intensity > grade II and hyperechoic area > 0.25 cm^2^ on TCS as level A evidence for differentiating primary PD from atypical Parkinson’s syndrome, for early diagnosis of PD, and for screening patients at high risk for PD (52). A study on the diagnostic value of PD by Tao et al. (70) showed that the sensitivity and specificity of SN hyperechogenicity for PD were 84 and 85%, respectively. The sensitivity and specificity to distinguish PD from healthy people were 85 and 89%, respectively. The sensitivity and specificity to distinguish PD from other Parkinsonian’s syndromes were 82 and 74%, respectively. Our study, involving 97 patients with PD and 56 controls, found that TCS was 88.7% sensitive and 82.1% to the diagnosis of PD.

In conclusion, TCS has become an important auxiliary detection method for PD diagnosis and differential diagnosis due to its advantages of convenience, rapid detection, invasiveness, low cost, high reproducibility, and easy promotion. However, the mechanisms by which SN hyperechogenicity occurs, the factors that influence it, and its clinical relevance to TCS are not fully understood and therefore need to be investigated in more detail.

While TCS offers several advantages, such as being non-invasive, relatively low cost, and having the ability to provide immediate results, its limitations must be carefully considered. These include dependence on operator skills, challenges in image interpretation, limitations in the availability of acoustic windows, and issues with resolution and specificity. For clinical and research applications, these limitations suggest that TCS should be used in conjunction with other diagnostic tools to provide a more comprehensive assessment of neurological conditions. Future technological advances and standardized training may help mitigate some of these limitations, enhancing the reliability and applicability of TCS in clinical neurology.

## Author contributions

Y-yZ: Conceptualization, Project administration, Writing – original draft, Writing – review & editing. X-hJ: Conceptualization, Project administration, Writing – original draft, Writing – review & editing. P-pZ: Data curation, Formal analysis, Writing – review & editing. W-yZ: Investigation, Methodology, Writing – review & editing. L-bL: Conceptualization, Project administration, Writing – original draft, Writing – review & editing.
